# Association between vitamin D3 levels and frailty in the elderly: A large sample cross-sectional study

**DOI:** 10.3389/fnut.2022.980908

**Published:** 2022-09-27

**Authors:** Zitian Zheng, Wennan Xu, Fei Wang, Yudian Qiu, Qingyun Xue

**Affiliations:** ^1^Department of Orthopedics, Beijing Hospital, National Center of Gerontology, Institute of Geriatric Medicine, Chinese Academy of Medical Sciences, Beijing, China; ^2^Peking University Fifth School of Clinical Medicine, Beijing, China; ^3^Department of Orthopedics, Sun Yat-sen Memorial Hospital, Sun Yat-sen University, Guangzhou, China

**Keywords:** disability, endocrinology, vitamin D, 25-hydroxyvitamin D3, frailty

## Abstract

**Background:**

Frailty is recognized as a cornerstone of geriatric medicine. Accurately screening and identifying frailty can promote better quality and personalized medical services for the elderly. Previous studies have shown that the association between vitamin D and frailty in the elderly population is still controversial. More research is needed to explore the association between them.

**Materials and methods:**

We used three waves of data from the National Health and Nutrition Examination Survey (NHANES). Based on the widely accepted AAH FRAIL Scale, we measured and evaluated the participants’ frailty from five aspects: fatigue, resistance, ambulation, illness, and loss of weight. All possible relevant variables are included. Machine learning XGboost algorithm, the Least Absolute Shrinkage Selection Operator (LASSO) regression and univariate logistic regression were used to screen variables, and multivariate logistic regression and generalized additive model (GAM) were used to build the model. Finally, subgroup analysis and interaction test were performed to further confirm the association.

**Results:**

In our study, XGboost machine learning algorithm explored the relative importance of all included variables, which confirmed the close association between vitamin D and frailty. After adjusting for all significant covariates, the result indicated that for each additional unit of 25-hydroxyvitamin D3, the risk of frailty was reduced by 1.3% with a statisticaldifference. A smooth curve was constructed based on the GAM. It was found that there was a significant negative correlation between 25-hydroxyvitamin D3 and the risk of frailty.

**Conclusion:**

There may be a negative correlation between 25-hydroxyvitamin D3 and the risk of frailty. However, more well-designed studies are needed to verify this relationship.

## Introduction

In the past few decades, with the rapid progress of medicine, life expectancy has increased significantly worldwide (1). As a result, the number of the elderly has increased, and it is expected that the population over the age of 60 will double in the next 30 years (2). However, because of the heterogeneity of human beings and the complexity of social economy and environment (3, 4), human aging is often heterogeneous (5). Therefore, scientists introduced the concept of frailty to better understand the heterogeneity of human aging (6).

Frailty is an age-related clinical condition, which is characterized by the reduction of individual homeostasis reserve and excessive vulnerability to endogenous and exogenous stressors (7, 8). This negative health condition between health and disability will lead to a disproportionate increase in adverse medical risk events relative to risk exposure factors (9). Frailty is favored by researchers because its contribution to the improvement of the traditional health care system largely conforms to the medical needs of the rapidly aging society (10). It is worth noting that frailty can be reversed (11). Accurately screening and identifying frail people or people who may develop into frailty is one of the key factors to provide them with high-quality medical treatment and care (12).

Frailty has a higher incidence rate in the elderly, and is more directly related to long-term nutritional status (13). The frailty of young people is often caused by major diseases and trauma, which is less related to nutrition (8, 14). Previous studies have shown that the prevalence of frailty among the elderly over 65 years old in the community is 10–20%, while the prevalence of frailty among the elderly over 85 years old in the community has risen to 30–45% (15).

In the past 30 years, the criteria for screening and identifying frailty have not been agreed. In several recent studies, it has been confirmed that there is no significant difference in prediction accuracy between frailty measurement tools such as Fried Phenotype, Edmonton FRAIL Scale and AAH Frailty Index (16). AAH FRAIL Scale, which has been widely proved to be efficient, was selected as the measure of frailty in our study (17).

Vitamin D has many functions, such as promoting the absorption of calcium and phosphorus, cell growth and differentiation, and regulating immune function (18, 19). 25-hydroxyvitamin D3 is the main circulating form of vitamin D, and because of its stable nature and long half-life, it is regarded as the is the best indicator of vitamin D level in the body (20). The activated form of vitamin D, 1,25-dihydroxyvitamin D3, has been shown to induce monocytes to differentiate into macrophages and reduce the release of inflammatory cells and chemokines (19). The differentiation and aging of cells, the level of immunity and inflammation in the body will affect the health status of the human body and the individual internal balance ability reserve to varying degrees.

Driven by the rapid development of computer processing power, memory and storage, machine learning algorithms are trained to efficiently obtain the required information from massive data (21). XGboost is an efficient implementation of the Gradient Boosting Decision Tree (GBDT) algorithm (22), which further improves the accuracy of intelligent prediction, avoids overfitting, improves the generalization of the algorithm, and further enhances the interpretability (23), thus becoming a widely accepted algorithm in machine learning and data mining (24). For the establishment of XGboost model and LASSO regression algorithms (25), we use the 10-fold cross validation method to obtain the model performance of the entire dataset (26). For cross validation, the dataset was divided into 10 folds, of which onefold was used as the test set and the rest as the training set; All the results of the 10 repetitions were taken as the average of the overall performance (27). According to the results, the optimal machine learning coefficients are selected.

## Materials and methods

### Data source

The present study analyzed respondent data from the National Health and Nutrition Examination Survey (NHANES), which was collected in three cycles (2007–2008, 2009–2010, and 2013–2014) by the Centers for Disease Control and Prevention (CDC), National Center for Health Statistics (NCHS) in the USA.

### Ethical considerations

All patient information in the database is anonymous, and all participants are aware of and consent to the data collection activities. The NHANES program ethical approval and informed consent signed by participants were obtained before NHANES collected data. No further ethical approval and informed consent are required for this study.

### Study population

The data of NHANES database from the three cycles was selected. A total of 30,861 participants took the survey. Protocols used in the NHANES were approved by US National Center for Health Statistics Research Ethics Review Board, and written informed consent was provided by all participants.

According to the definition of the elderly by the United Nations and World Health Organization,^1, 2^ we included participants older than 65 years old.

The inclusion and exclusion criteria were as follows: (1) No lack of relevant data of all the indicators of the previously validated FRAIL Scale (Appendix 1). FRAIL Scale items in AAH) (2) No lack of data of relevant biochemical indexes such as serum vitamin D3 levels and all other biochemical covariates (see below). (3) No lack of data of general demographic characteristics of the participants, including race, age, income, use of alcohol and tobacco. (4) According to the standards of the World Health Organization and United Nations, people over 65 years old are defined as the elderly. Finally, a total of 527 participants were included in the study.

### Study variables

The variables of each case were included in the study: (1) Patient demographics: gender, age, race, income, education level (2) Variables related to the FRAIL Scale index, including relevant questionnaire data related to fatigue, resistance, ambulation, illness, loss of weight (3) Relevant laboratory examination indicators: including urine albumin, urine creatinine, serum albumin, alanine aminotransferase (ALT), aspartate aminotransferase (AST), alkaline phosphatase (ALP), blood urea nitrogen (BUN), total calcium, cholesterol, serum creatinine, gamma glutamyl transferase (GGT), serum glucose, refrigerated serum iron, lactate dehydrogenase (LDH), phosphorus, total bilirubin, total protein, uric acid, sodium, potassium, chloride, globulin, high-density lipoprotein (HDL), low-density lipoprotein (LDL), triglyceride and 25-hydroxyvitamin D3. (4)The participants’ body mass index (BMI), defined as the weight divided by the square of height (kg/m^2^)(28). Finally, we included a total of 57 variables.

### Evaluation criterion

#### Frailty

All included subjects were categorized into robust (scored 0), pre-frail (scored 1–2) and frail (scored 3–5) clusters according to the previously validated FRAIL Scale. A complete description of the FRAIL Scale items scoring criteria, and baseline prevalence are provided in Appendix 1 (29).

#### Hypertension

The average value of three blood pressure measurements was calculated, and the mean blood pressure was used to assess whether the participants are hypertensive. The diagnostic criteria of hypertension are systolic pressure ≥ 140 mmHg and/or diastolic pressure ≥ 90 mmHg.

#### Drinking

We examined the classification of alcohol consumption in previous studies, which was finally divided into two levels. An alcoholic is defined as a person who drinks more than 12 drinks per year.

### Vitamin D

Serum 25-hydroxyvitamin D3 concentrations were measured at the National Center for Environmental Health, CDC, Atlanta, GA using the DiaSorin RIA kit (Stillwater MN). Serum 25-hydroxyvitamin D3 status was classified into three levels: Participants with serum 25-hydroxyvitamin D3 < 30 nmol/L were defined as deficiency; Participants with concentrations > 30 but < 50 nmol/L were defined as insufficiency; Participants with concentrations > 50 nmol/L were considered as normal vitamin D status (30).

### Statistical analysis

*T*-test, Mann–Whitney U test and weighted linear regression were used for continuous variables and Chi-square tests were used for categorical variables. LASSO regression was employed to screen variables, and multivariate logistic regression model was developed to analyze the significance of variables. The generalized additive model (GAM) can better fit the association between vitamin D3 and frailty and avoid the possibility of overfitting and underfitting (31, 32). Finally, we performed subgroup analysis in the presence of interaction effects for further exploration.

In addition, the XGboost algorithm is used to calculate, rank and output the most relevant and significant variables of the state of frailty of the participants, so as to verify the stability and reliability of our conclusions. We tested the collinearity in the regression model by calculating variance inflation factors (VIF) and excluded independent variables with VIF > 10.

All analyses were completed by using STATA version 15 (StataCorp LP, College Station, TX, USA) and R statistical software (R4.0.2).

## Result

### Characteristics of study population

**FIGURE 1:**
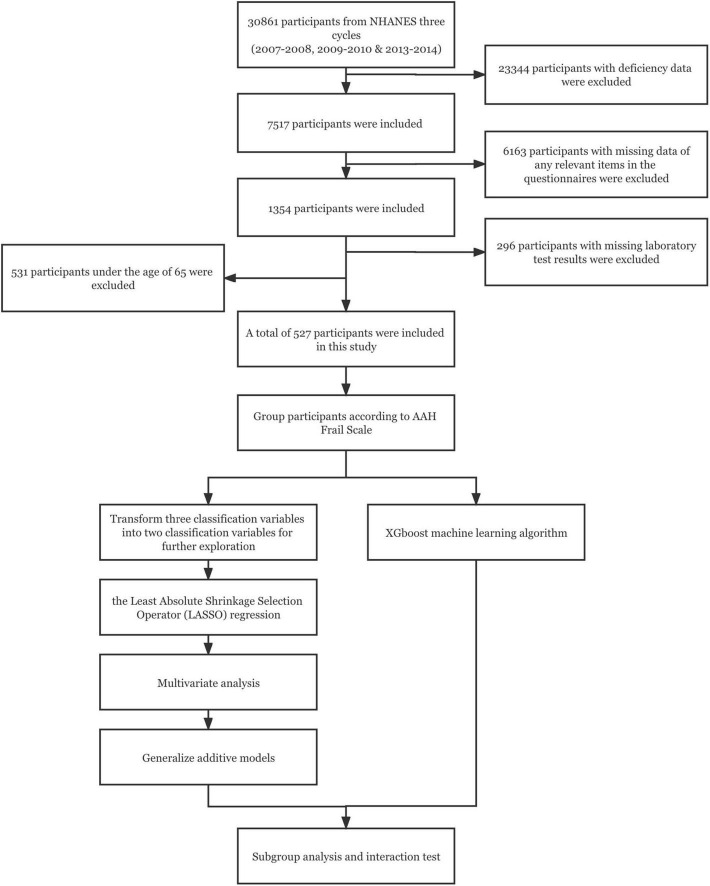
The work flow diagram.

In this study, 30,861 participants were obtained from the NHANES database. After excluding 23,344 participants with deficiency data, there were 7,517 participants including all the data items we needed. The 6,163 participants with missing data of any relevant items in the questionnaires (“Medical Conditions,” “Physical Functioning,” “Osteoporosis,” “Mental Health-Depression Screener,” “Alcohol Use,” “Smoking and Weight History” questionnaires) and the 296 participants with missing laboratory test results were excluded. Because this study focused on the health management of the elderly, 531 participants under the age of 65 were excluded according to the definition of the elderly by WHO, and 527 participants were finally included in this trial (Figure 1).

The age, education level and income of the participants in the robust, pre-frail and frail groups were found to be different (Table 1). The average age of the frail patients was significantly higher than the average age of the patients in the pre-frail group (*p* < 0.001), and was significantly higher than the average age of the participants in the robust group (*p* < 0.001). There was no significant difference in gender and race among the three groups according to the Chi-square test (Table 1).

**TABLE 1:** Candidate variables and baseline characteristics of the participants.

Variables	Total	Robust	Pre-frail	Frail	*P*-value	*P*-value*	*P*-value**	*P*-value***
**Sex**					0.152	0.935	0.063	0.060
Male	338 (64.1%)	170 (65.1%)	148 (65.5%)	20 (50.0%)				
Female	189 (35.9%)	91 (34.9%)	78 (34.5%)	20 (50.0%)				
**Age**	72.2 ± 5.0	71.8 ± 4.7	72.1 ± 5.1	75.2 ± 5.3	< 0.001	0.452	< 0.001	< 0.001
**Race**					0.508	0.228	0.536	0.211
Mexican American	46 (8.7%)	23 (8.8%)	22 (9.7%)	1 (2.5%)				
Non-Hispanic Black	36 (6.8%)	14 (5.4%)	20 (8.8%)	2 (5.0%)				
Non-Hispanic White	356 (67.6%)	177 (67.8%)	149 (65.9%)	30 (75.0%)				
Other Hispanic	73 (13.9%)	37 (14.2%)	29 (12.8%)	7 (17.5%)				
Other race	16 (3.0%)	10 (3.8%)	6 (2.7%)	0 (0.0%)				
**Education**					0.152	0.217	0.042	0.175
Non-received higher education	312 (59.2%)	170 (65.1%)	148 (65.5%)	20 (50.0%)				
Received higher education	215 (40.8%)	91 (34.9%)	78 (34.5%)	20 (50.0%)				
**Income**					0.049	0.131	0.024	0.153
Earning less than $1000,000	485 (92.0%)	234 (89.7%)	211 (93.4%)	40 (100.0%)				
Earning more than or equal to $1000,000	42 (8.0%)	27 (10.3%)	15 (6.6%)	0 (0.0%)				
**Smoking**					< 0.001	0.001	< 0.001	0.082
Every day	74 (14.0%)	20 (7.7%)	43 (19.0%)	11 (27.5%)				
Some days	11 (2.1%)	6 (2.3%)	3 (1.3%)	2 (5.0%)				
Not at all	442 (83.9%)	235 (90.0%)	180 (79.6%)	27 (67.5%)				
**Alcohol use**					0.532	0.394	0.359	0.648
Yes	431 (81.8%)	218 (83.5%)	182 (80.5%)	31 (77.5%)				
No	96 (18.2%)	43 (16.5%)	44 (19.5%)	9 (22.5%)				
BMI (kg/M^2^)	28.0 ± 5.3	27.2 ± 4.5	28.8 ± 5.6	28.3 ± 6.7	0.002	0.001	0.209	0.550
Albumin, urine (μg/mL)	75.4 ± 445.8	42.5 ± 172.2	65.9 ± 240.1	343.5 ± 1438.2	< 0.001	0.559	< 0.001	< 0.001
Creatinine, urine (mg/dL)	113.1 ± 65.2	110.9 ± 64.2	114.5 ± 66.0	120.2 ± 67.2	0.647	0.543	0.403	0.614
ALT (U/L)	22.2 ± 18.9	21.3 ± 11.1	23.6 ± 26.1	20.4 ± 8.9	0.332	0.179	0.785	0.326
AST (U/L)	25.4 ± 15.3	24.6 ± 8.1	26.8 ± 21.5	23.1 ± 7.3	0.172	0.111	0.569	0.160
GGT (U/L)	27.3 ± 26.5	24.5 ± 22.8	29.7 ± 28.7	32.0 ± 33.7	0.047	0.029	0.092	0.608
LDH (U/L)	136.6 ± 27.4	136.0 ± 25.2	136.4 ± 30.1	141.0 ± 26.3	0.556	0.864	0.281	0.328
ALP (U/L)	70.3 ± 24.0	69.0 ± 24.8	70.2 ± 21.5	79.5 ± 29.8	0.034	0.557	0.009	0.024
BUN (mmol/L)	5.8 ± 2.5	5.6 ± 2.0	5.7 ± 2.4	7.4 ± 4.4	< 0.001	0.545	< 0.001	< 0.001
Total calcium (mmol/L)	2.4 ± 0.1	2.4 ± 0.1	2.4 ± 0.1	2.4 ± 0.1	0.693	0.590	0.437	0.628
Creatinine, serum (μmol/L)	90.0 ± 42.5	86.3 ± 23.3	88.7 ± 27.6	121.9 ± 123.2	< 0.001	0.525	< 0.001	< 0.001
Glucose, serum (mmol/L)	6.1 ± 1.9	6.0 ± 1.8	6.1 ± 1.9	6.7 ± 2.1	0.149	0.595	0.051	0.099
Chloride (mmol/L)	103.5 ± 3.3	103.7 ± 3.2	103.4 ± 3.1	103.4 ± 4.2	0.682	0.395	0.684	0.962
Iron, refrigerated (μmol/L)	15.9 ± 5.5	16.5 ± 5.5	15.7 ± 4.8	13.2 ± 8.0	0.001	0.092	< 0.001	0.007
Phosphorus (mmol/L)	1.2 ± 0.2	1.2 ± 0.2	1.2 ± 0.2	1.2 ± 0.2	0.046	0.951	0.015	0.018
Uric acid (μmol/L)	352.0 ± 86.3	347.0 ± 83.8	355.7 ± 83.6	363.7 ± 113.7	0.364	0.269	0.254	0.587
Sodium (mmol/L)	139.6 ± 2.5	139.6 ± 2.6	139.6 ± 2.5	139.9 ± 1.9	0.681	0.767	0.383	0.480
Potassium (mmol/L)	4.1 ± 0.4	4.1 ± 0.4	4.1 ± 0.4	4.2 ± 0.5	0.596	0.554	0.512	0.336
Albumin, serum (g/L)	139.6 ± 2.5	41.9 ± 2.7	41.8 ± 2.8	40.8 ± 3.6	0.057	0.549	0.017	0.040
Globulin, serum (g/L)	29.2 ± 4.9	29.1 ± 4.7	29.2 ± 4.8	30.1 ± 5.8	0.426	0.828	0.193	0.241
HDL-Cholesterol (mmol/L)	1.4 ± 0.4	1.5 ± 0.4	1.4 ± 0.4	1.4 ± 0.3	0.142	0.057	0.321	0.980
Triglyceride (mmol/L)	1.4 ± 0.7	1.3 ± 0.6	1.5 ± 0.8	1.4 ± 0.7	0.071	0.022	0.588	0.495
LDL-cholesterol (mmol/L)	2.8 ± 1.0	2.9 ± 1.0	2.7 ± 1.0	2.4 ± 0.9	0.007	0.045	0.004	0.078
25-hydroxyvitamin D3 (nmol/L)	65.7 ± 28.0	70.0 ± 27.9	62.2 ± 27.5	57.1 ± 27.1	0.001	0.002	0.006	0.284

% for: Sex Race Education Income Smoking Alcohol-use. *P*-value was calculated by Chi-square test. Mean ± SD for: Age BMI (kg/m^2^) Albumin, urine (μg/mL); Creatinine, urine (mg/dL); ALT (U/L); AST (U/L); ALP (U/L); BUN (mmol/L); Total calcium (mmol/L); Creatinine, serum (μmol/L) GGT (U/L); Glucose, serum (mmol/L); Iron, refrigerated (μmol/L) LDH (U/L); Phosphorus (mmol/L); Uric acid (μmol/L); Potassium (mmol/L) Chloride (mmol/L); Globulin, serum (g/L); HDL-Cholesterol (mmol/L); Triglyceride (mmol/L); LDL-cholesterol (mmol/L) Albumin, serum (g/L) Sodium (mmol/L); 25-hydroxyvitamin D3 (nmol/L). *P*-value was calculated by weighted linear regression model. *P*-value*: *P*-value for Robust-Pre-frail based on Fisher’s Least Significant Difference (LSD) *post hoc* test. *P*-value**: *P*-value for Robust-Frail based on Fisher’s Least Significant Difference (LSD) *post hoc* test. *P*-value***: *P*-value for Pre-frail-Frail based on Fisher’s Least Significant Difference (LSD) *post hoc* test. BMI, body mass index; ALT, alanine aminotransferase; AST, aspartate aminotransferase; ALP, alkaline phosphatase; BUN, blood urea nitrogen; GGT, gamma glutamyl transferase; LDH, lactate dehydrogenase; HDL, high-density lipoprotein; LDL, low-density lipoprotein.

In addition, it was observed that there were significant differences in BMI, urinary albumin, ALP, BUN, serum creatinine, serum glucose, phosphorus, total protein, serum albumin, serum globulin and 25-hydroxyvitamin D3 levels among the three groups according to the weighted linear regression model. The average BMI of the pre-frail participants was significantly higher than that of the robust participants (*p* = 0.001), but participants with frailty were no different from other two groups in terms of BMI. However, the urinary albumin, BUN and serum creatinine in the frail group were significantly higher than those in the pre-frail group and the robust group. This demonstrated that the frailty of the elderly is closely related to renal function, and the inclusion and adjustment of serum biomarkers related to chronic kidney disease will greatly optimize our analysis. In addition, there were also significant differences in biomarkers related to systemic nutritional status and liver disease among the three groups. Serum albumin in the frail group was lower and globulin was higher than that in the pre-frail group and the robust group. There was no significant difference in the concentration triglycerides, LDL-cholesterol, HDL-Cholesterol and aminotransferases among the three groups (Table 1).

### Single factor analysis: Univariate logistic regression

In order to further explore the relationship between 25 hydroxyvitamin D3 level and the occurrence of frailty, we included the robust group and the pre-frailty group into the non-frailty group, which corresponds to the frailty group.

Univariate logistic regression was performed to analyze the significance of associations between the occurrence of frailty and the serum levels of 25-hydroxyvitamin D3, other laboratory indicators, gender, age, race, education level, income level, and BMI. The serum levels of 25-hydroxyvitamin D3, iron, HDL cholesterol, LDL cholesterol and total cholesterol were negatively correlated with the occurrence of frailty, while the serum levels of GGT, serum creatinine, triglycerides and BMI were positively correlated with the occurrence of frailty. In addition, probability of frailty was higher in the low-income population and smoking population (Table 2).

**TABLE 2:** Univariate analysis for frailty.

Univariate logistic regression		
Variables	OR (95% CI)	*P*-value
**Sex**		
Male	1	
Female	1.090 (0.763, 1.556)	0.636
**Age**	1.033 (0.998, 1.069)	0.066
**Race**		
Mexican American	1	
Non-Hispanic White	1.571 (0.649, 3.807)	0.316
Non-Hispanic Black	1.011 (0.547, 1.869)	0.971
Other Hispanic	0.973 (0.465, 2.035)	0.941
Other race	0.600 (0.187, 1.925)	0.390
**Education**		
Non-received higher education	1	
Received higher education	0.741 (0.523, 1.050)	0.091
**Income**		
Earning less than $1000,000	1	
Earning more than or equal to $1000,000	0.518 (0.269, 0.998)	0.049
**Smoking**		
Every day	1	
Some days	0.309 (0.085, 1.125)	0.074
Not at all	0.326 (0.189, 0.563)	< 0.001
**Alcohol use**		
Yes	1	
No	1.261 (0.809, 1.967)	0.306
BMI (kg/m^2^)	1.060 (1.025, 1.097)	< 0.001
Albumin, urine (μg/mL)	1.001 (1.000, 1.002)	0.107
Creatinine, urine (mg/dL)	1.001 (0.998, 1.004)	0.432
ALT (U/L)	1.007 (0.993, 1.020)	0.319
AST (U/L)	1.009 (0.993, 1.026)	0.258
GGT (U/L)	1.010 (1.001, 1.018)	0.024
LDH (U/L)	1.001 (0.995, 1.008)	0.639
ALP (U/L)	1.005 (0.997, 1.012)	0.203
BUN (mmol/L)	1.067 (0.994, 1.145)	0.074
Total calcium (mmol/L)	2.000 (0.293, 13.660)	0.479
Creatinine, serum (μmol/L)	1.006 (1.000, 1.013)	0.048
Glucose, serum (mmol/L)	1.051 (0.957, 1.153)	0.299
Chloride (mmol/L)	0.977 (0.927, 1.029)	0.381
Iron, refrigerated (μmol/L)	0.960 (0.930, 0.991)	0.012
Phosphorus (mmol/L)	1.522 (0.518, 4.473)	0.445
Uric acid (μmol/L)	1.001 (0.999, 1.003)	0.188
Sodium (mmol/L)	1.018 (0.951, 1.090)	0.603
Potassium (mmol/L)	0.928 (0.598, 1.440)	0.739
Total protein (g/L)	0.998 (0.965, 1.031)	0.887
Globulin, serum (g/L)	1.010 (0.975, 1.047)	0.565
Albumin, serum (g/L)	0.963 (0.907, 1.023)	0.218
HDL-Cholesterol (mmol/L)	0.658 (0.433, 0.999)	0.049
Triglyceride (mmol/L)	1.326 (1.029, 1.710)	0.029
LDL-cholesterol (mmol/L)	0.791 (0.661, 0.945)	0.009
Total Cholesterol (mmol/L)	0.836 (0.718, 0.973)	0.020
Bilirubin, total (μmol/L)	0.996 (0.960, 1.033)	0.816
25-hydroxyvitamin D3 (nmol/L)	0.989 (0.983, 0.995)	< 0.001

BMI, body mass index; ALT, alanine aminotransferase; AST, aspartate aminotransferase; ALP, alkaline phosphatase; BUN, blood urea nitrogen; GGT, gamma glutamyl transferase; LDH, lactate dehydrogenase; HDL, high-density lipoprotein; LDL, low-density lipoprotein.

### Multivariate analysis: Least Absolute Shrinkage Selection Operator regression

As is shown in Supplementary Table 1, we tested the collinearity in the regression model and excluded three variables: cholesterol, total bilirubin, and total protein.

**FIGURE 2:**
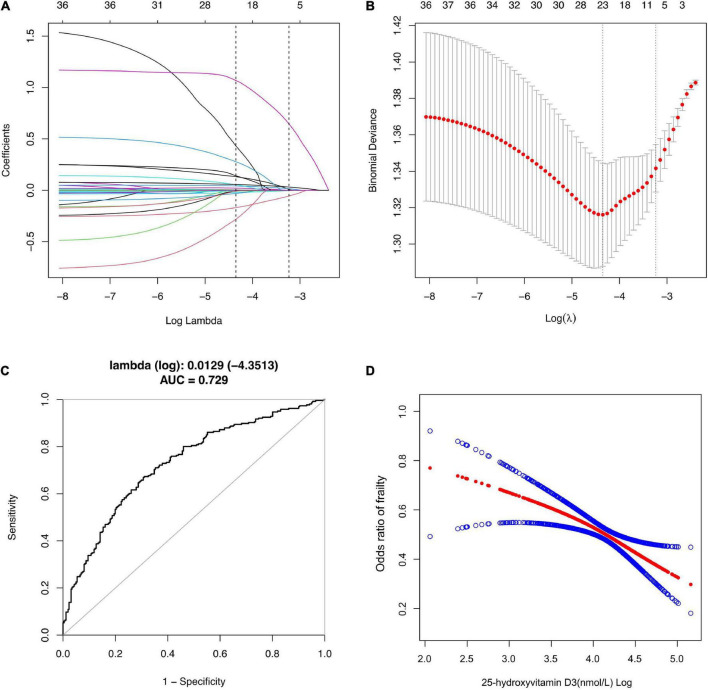
Selection of Variables using LASSO regression. **(A)** Lasso coefficient of 31 variables in model 1; **(B)** the optimal penalty coefficient [Lambda (log) = 0.0129] in the Lasso regression was identified with the minimum criterion; **(C)** Receiver operating characteristic (ROC) curves according to LASSO Regression; **(D)** The relationship between Vitamin D3 and Frailty. Solid rad line represents the smooth curve fit between variables according to GAM. Blue bands represent the 95% of confidence interval from the fit.

Figure 2 depicted the results of selection of variables by using LASSO regression. In Figure 2B, the red dots indicate the tuning parameter (Lambda) and the two dotted lines represent the two special Lambda (log) values selected in the LASSO model using 10-fold cross-validation, namely, value of lambda (log) that gives minimum mean cross validated error [Lambda. min (log)] and large value of lambda (log) such that error is within 1 standard error of the minimum [Lambda.1se (log)]. The variables selected by these two lambda values are the variables included in the corresponding optimization model. The Lambda min (log) was 0.0129 (-4.3513). The AUC value of Receiver Operating Characteristic (ROC) curves of the prediction model based on the variables screened by the Lambda values are 0.729 (Figure 2C), indicating that LASSO regression can effectively screened out the variables with strong correlation with frailty.

According to the Lambda min (log) of 10-fold cross-validation, the selected variables are urine albumin, AST, BUN, total calcium, serum creatinine, GGT, refrigerated serum iron, chloride, triglyceride, LDL-cholesterol, serum globulin, sodium, age, race, income, smoking, alcohol use, BMI and 25-hydroxyvitamin D3 (Supplementary Table 2).

The variables screened by LASSO regression were included into the multivariate logistic regression. In the fully adjusted model, it was found that the risk of frailty will be reduced by 1.3% for each additional unit of 25-hydroxyvitamin D3.

For sensitivity analysis, we transformed 25-hydroxyvitamin D3 from continuous variable to classified variables—sufficiency, insufficiency, and deficiency. The *P*-value of 25-hydroxyvitamin D3 trend in the two model is consistent with the result when 25-hydroxyvitamin D3 is a continuous variable. The risk for frailty was 1.5 and 2.2 times higher in vitamin D insufficient and deficient group, respectively, compared with vitamin D sufficient group (Table 3).

**TABLE 3:** The association between VitD3 and Frailty in the multiple regression model.

Multivariate analysis		
Variables	OR (95% CI)	*P*-value
Model 1	0.989 (0.983, 0.995)	< 0.001
Model 2	0.989 (0.982, 0.996)	0.002
Model 3	0.987 (0.979, 0.995)	< 0.001
25-hydroxyvitamin D3 status		
Sufficiency	Reference	
Insufficiency	1.506 (0.920, 2.466)	
Deficiency	2.244 (1.119, 4.497)	
*P* for trend	0.011	

Model 1: No adjustments have been made; Model 2: Adjustments were made for gender, age, race, smoking, alcohol use, income, and education level; Model 3: Adjustments were made for age, race, income, smoking, alcohol use, BMI, urine albumin, AST, BUN, serum creatinine, refrigerated iron, chloride, sodium, globulin, triglyceride, and LDL-cholesterol according to LASSO regression. BMI, body mass index; ALT, alanine aminotransferase; AST, aspartate aminotransferase; ALP, alkaline phosphatase; BUN, blood urea nitrogen; GGT, gamma glutamyl transferase; LDH, lactate dehydrogenase; HDL, high-density lipoprotein; LDL, low-density lipoprotein.

### Generalized additive model

According to our sensitivity test, the correlation between vitamin D3 as a categorical variable and frailty is not completely consistent with the correlation between vitamin D3 as a continuous variable. Linear regression better describes the association between two continuous variables while controlling for other confounders, compared with logistic regression, but in case there in non-linear association, GAM is more applicable (33). GAM is a statistical model that fits data with a higher degree of freedom (34). Before modeling, it is not necessary to analyze the relationship between response variables and explanatory variables. Instead, the response variables and each explanatory variable are modeled separately and added to obtain GAM (35). After adjusting the statistically significant variables screened by LASSO regression in GAM, it was found that the level of 25-hydroxyvitamin D3 was significantly negatively correlated with the risk of frailty (Figure 2D).

### Subgroup analysis

**FIGURE 3:**
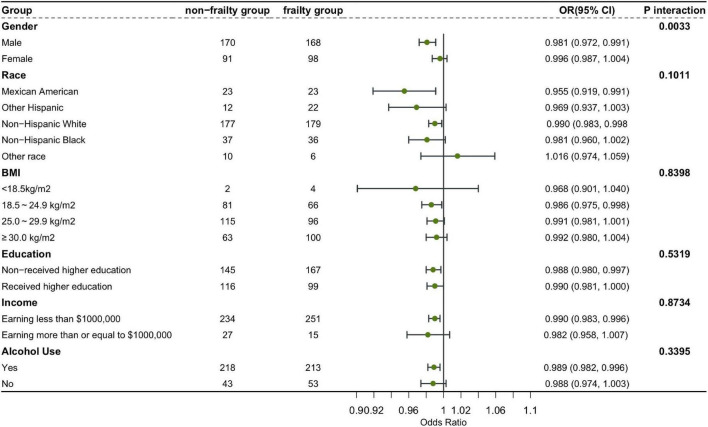
Subgroup analysis and interaction test.

In order to better explain this result, we conducted subgroup analysis and interaction test (36). This study, stratified by gender, race, education level, income, BMI and alcohol use, verified whether the conclusion of the relationship between 25-hydroxyvitamin D3 level and the risk of frailty based on the overall population was still applicable to each subgroup. The results showed that except for the interaction between 25-hydroxyvitamin D3 and the risk of frailty in the sex subgroup of model 1, there was no interaction in other subgroups. Our conclusion is stable and reliable (Figure 3).

### Machine learning using the XGBoost algorithm model

**FIGURE 4:**
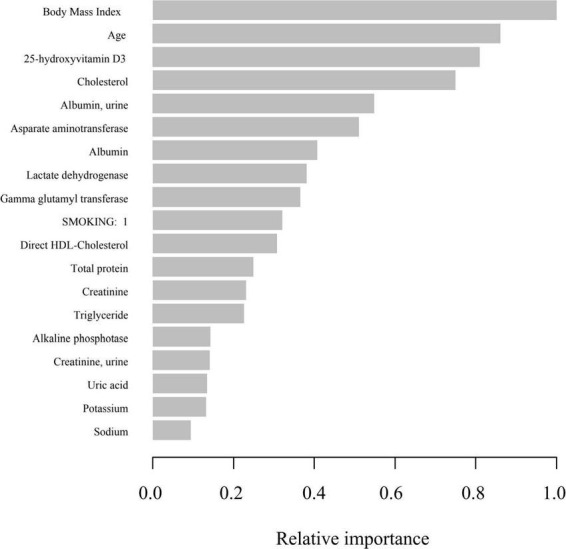
Relative importance of the selected variables using XGBoost and the corresponding variable importance score. *X*-axis indicates the importance score, which is the relative number of a variable that is used to distribute the data, *Y*-axis indicates the selected variable.

In the development and validation phase of the research, we input all the variables into the XGboost machine learning algorithm based on 10-fold cross validation method for the three classifications of states of frailty. These variables include sociodemographic data of participants and all relevant laboratory data. According to the relative importance of variables attached by XGboost algorithm, BMI, age, 25-hydroxyvitamin D3, cholesterol and urine albumin are the five most significant variables in the dataset (Figure 4).

## Discussion

With the development of population aging, more and more elderly people are in a state defined as frailty due to the impairment of their physical and psychological functions, which has brought a heavy burden to individuals, families and society (5, 37). The frailty of the elderly is due to the cumulative decline of physiological function and the increase of physical and mental vulnerability related to aging, which reduces the ability of the elderly to effectively cope with diseases or trauma, and will lead to more adverse consequences: falls, delirium, incontinence, etc. (38).

The relationship between vitamin D and health has been a long-discussed topic (39, 40). In recent years, the relationship between vitamin D and frailty in the elderly has gradually attracted researchers’ attention (41). Through in-depth analysis of the data of the U.S. National Health and Nutrition Examination Survey, this study shows that 25-hydroxyvitamin D3 is an important protective factor for the frailty of the elderly. Vitamin D can affect the body function through several mechanisms. Firstly, it can affect the body function through the potential mechanism of indirectly regulating calcium and phosphorus metabolism. Secondly, it can directly regulate the transcription of genes related to calcium channels (42) and skeletal muscle proliferation and differentiation at the genome level by combining with vitamin D receptor (VDR) on skeletal muscle cells (43, 44).

Frailty also has a great impact on the perioperative period (45). In many clinical trials, it has been found that preoperative frailty and low serum 25 hydroxyvitamin D3 can directly increase the probability of postoperative mortality and serious complications, predicting a worse prognosis (46–48). In addition, Jim Bartley has shown that vitamin D can reduce the probability of postoperative pulmonary infection through anti-inflammatory effect (49). The above findings prove that it is beneficial to supplement appropriate amount of vitamin D during perioperative period. The identification and mitigation of patients’ frailty is of great reference value to anesthesiology and surgery (50, 51). Our results confirmed that the level of 25-hydroxyvitamin D3 was negatively correlated with the occurrence of frailty, which can be used in clinical practice to improve the prognosis of patients with frailty.

The subgroup and interaction analysis confirmed that our conclusion is robust and reliable. Subgroup analysis showed that women were more likely to become frail than men, non-Hispanic Whites, people with higher education and low-income groups were more likely to be frail. Gender may affect the expression of aging related genes and the epigenetic changes of aging (52), including mitochondrial dysfunction in aging (53). According to the research of Therri Usher et al. the difference of frailty between races cannot be explained by obesity, chronic disease burden or low-income people (54). More research on racial frailty needs to be carried out to implement targeted interventions. High-income groups may reduce the probability of frailty because of better lifestyle and better medical conditions (55, 56). In addition, it was found that the risk of frailty increased with the increase of BMI. However, according to previous meta-analysis (57), frailty is positively associated with both underweight and obesity. This may be because our analysis is based on the sample from the United States. As a developed economy, the United States has a high obesity rate (58), and there is also a certain interaction between BMI and household income level (59). It may be difficult for lower income people to maintain healthier living habits, which leads to weakness to a certain extent. In addition, our sample of people with lower BMI is also small, which may also lead to some bias.

Compared with previous studies, our study has certain advantages and innovation. Firstly, our study was based on the real-world population study in the United States, and 527 elderly people over 65 years old are included, forming a cross-sectional study with a large sample size. Secondly, we adopted XGboost algorithm and LASSO regression which have been proved to be extremely robust and efficient to screen and verify variables. Additionally, to avoid overfitting and underfitting, we built the GAM to fit the results, which demonstrated the non-linear negative correlation between the possibility of frailty and 25-hydroxyvitamin D3 levels. Finally, we performed subgroup analysis and interaction test to verify the reliability of the conclusions and extend the application scope of our conclusion.

In addition, our research has certain limitations; (1) It is difficult to distinguish causal relationship through cross-sectional study, and we will further apply prospective cohort study to deepen the conclusion in the future; (2) The sample of this study is based on the population survey data of the United States, so whether the conclusions of this paper can be extended to the population in other countries and regions needs further research and investigation; (3) The elderly often have chronic diseases, including chronic heart disease, chronic kidney disease, etc. Our study has included and adjusted biomarkers related to chronic kidney disease, chronic liver disease and other chronic diseases to a certain extent, but there are still some biomarkers of diseases that may lead to frailty have not been included and adjusted. (4) It is worth noting that the assessment of frailty in this paper is based on the AHH Frailty Scale, which may lead to some bias. Among a variety of frailty screening tools, frailty scale and frailty index have been proved to have the strongest predictive validity for disability and mortality (60), and their test efficiency has been confirmed in a variety of populations, including middle-aged women (61), African American people, and Mexican adults (62). However, a number of comparative studies and cohort studies have shown that although there is moderate consistency and strong positive correlation between a variety of frailty screening tools (60, 61, 63), there are still some differences in the frailty incidence rate, and these differences will affect our results to a certain extent. In the future, we will apply multiple frailty screening tools based on NHANES database, including Fried Frailty Phenotype, Frailty Index, FRAIL Scale, Clinical Frailty Scale, Time Up-and-Go Test, Tilburg Frailty Indicator, Groningen Frailty Indicator and Edmonton Frailty Scale, to conduct comparative research to comprehensively demonstrate the consistency, correlation, and bias among the multiple frailty screening tools for predicting the frailty of the elderly.

## Conclusion

In conclusion, the results of this cross-sectional study based on the data of the U.S. National Health and Nutrition Examination Survey database show that there is a significant negative correlation between serum vitamin D3 levels and frailty in the elderly population. The confirmation and revelation of the negative correlation will be of great guiding significance to clinical work including perioperative management.

## Data availability statement

The raw data supporting the conclusions of this article will be made available by the authors, without undue reservation.

## Ethics statement

The studies involving human participants were reviewed and approved by the Protocols used in the NHANES were approved by US National Center for Health Statistics Research Ethics Review Board, and written informed consent was provided by all participants information on this is available on the official NHANES site: https://wwwn.cdc.gov/Nchs/Nhanes/ (https://www.cdc.gov/nchs/nhanes/irba98.htm). And the data for the explored NHANES surveys are publicly available for exploration (https://www.cdc.gov/nchs/data_access/restrictions.htm). The patients/participants provided their written informed consent to participate in this study.

## Author contributions

QX: project administration, validation data curation, and supervision. ZZ: conceptualization, methodology, data curation, formal analysis, and writing – original draft. WX, YQ, and FW: methodology and writing – review and editing. All authors contributed to the article and approved the submitted version.

## Footnotes

1
https://previous.iiasa.ac.at/web/home/about/news/190227-aging.html

2
https://previous.iiasa.ac.at/web/home/research/researchPrograms/WorldPopulation/Meetings/181105-UNDESA.html

## Supplementary material

Supplementary Table 1 Stepwise screening of collinearity of independent variables.

Supplementary Table 2 Coefficients assigned to variables according to LASSO regression.

Supplementary Table 3 Relative importance of the selected variables using XGBoost and the corresponding importance score of each variable.

## Appendix 1

FRAIL scale items in AAH

Fatigue: “How much of the time during the past 4 weeks did you feel tired?” 1 = All of the time, 2 = Most of the time, 3 = Some of the time, 4 = A little of the time, 5 = None of the time. Responses of “1” or “2” are scored as 1 and all others as 0. Baseline prevalence = 20.1%.

Resistance: “By yourself and not using aids, do you have any difficulty walking up 10 steps without resting?” 1 = Yes, 0 = No. Baseline prevalence = 25.5%.

Ambulation: “By yourself and not using aids, do you have any difficulty walking several hundred yards?” 1 = Yes, 0 = No. Baseline prevalence = 27.7%.

Illness: For 11 illnesses, participants are asked, “Did a doctor ever tell you that you have [illness]?” 1 = Yes, 0 = No. The total illnesses (0–11) are recoded as 0–4 = 0 and 5–11 = 1. The illnesses include hypertension, diabetes, cancer (other than a minor skin cancer), chronic lung disease, heart attack, congestive heart failure, angina, asthma, arthritis, stroke, and kidney disease. Baseline prevalence = 2.1%.

Loss of weight: “How much do you weigh with your clothes on but without shoes? [current weight]” “One year ago in (MO, YR), how much did you weigh without your shoes and with your clothes on? [weight 1 year ago]” Percent weight change is computed as: [(weight 1 year ago – current weight]/weight 1 year ago)] × 100. Percent change > 5 (representing a 5% loss of weight) is scored as 1 and < 5 as 0. Baseline prevalence = 21.0%.
